# Supplementary material for: Impact of Modern Lifestyle on Circadian Health and Its Contribution to Adipogenesis and Cancer Risk
Source: Cancers (Basel). 2024 Nov 1;16(21):3706. doi: 10.3390/cancers16213706 (PMC11545514; doi:10.3390/cancers16213706)
Supplement: Supplementary file 1 [file cancers-16-03706-s001.zip › Supplementary Table S1.pdf]

Table S1. Modern habits act as circadian disruptors affecting cortisol chronobiology.

Circadian Disruptor	Participants	Alteration	Comments	Reference
Shift-work	Security guards	Increase in CAR (15%)<br>Increase in CN (26%)	Cortisol levels were significantly higher in night-workers security guards and tended to increase if they were subjected to an operative task (e.g. Those monitoring the streets vs monitoring cameras).	[60]
	Midwives	Increase in CR (0.27-4-fold)<br>Loss of rhythmicity	Midwives on 3 months of night-shift work exhibit sustained cortisol levels (did not fluctuate), with higher levels at night	[272]
	Physicians	Increase in CAR (35%)	The shift work changes the diurnal cortisol pattern	[273]
	Pilots	Increase in CAR (20%)<br>Early shift in the acrophase<br>Reduced cortisol slope	Early shifts were associated with increased cortisol production and slower decline during the day.	[274]
	Permanent night-workers	Increase in CR during sleep time (2.3-fold)<br>Reduction in CR during active periods (2-fold)<br>Presence of irregular peaks<br>Flattening of CR	Permanent night workers exhibit disturbances in cortisol profiles	[58]
	Permanent night-workers	Increase in CR during night (57%)<br>Presence of irregular peaks during sleep time	Permanent night workers displayed a shift in cortisol production and additional peaks of cortisol during sleep time	[59]
Social Jetlag	University students and employees	Increase in CAR (29%)<br>Increase in CR (AUC)	Social Jetlag was associated with sleep disorders and increased cortisol levels during the day	[56]
	Healthy volunteers	Increase in CAR (10-42%)<br>Increase in CR (AUC)	Cortisol levels from 40 participants under social Jetlag were very heterogeneous but tend to increase in comparison to control participants.	[275]
Sleep-deprivation	Healthy volunteers	Increase in CR (AUC)<br>Presence of irregular peaks during sleep time	Acute sleep deprivation increased the cortisol levels during the night and the day after the night of sleep deprivation	[54]
	Healthy volunteers	Increase in CAR (37-45%)	Partial or total sleep deprivation significantly increases cortisol production (37 and 45% respectively).	[53]
	Healthy volunteers	Increase in basal cortisol (80%)<br>Increase in CR (AUC)	Sleep deprivation led to a higher cortisol baseline and amplified cortisol response to a stressor	[51]
	Healthy volunteers	Increase in CR (1.3-Fold)	Acute (24h) sleep deprivation significantly increased cortisol production	[52]

Light exposure	Healthy volunteers	Increase in CR (50%)<br>Persistent high cortisol	Acute bright light (1240 Lux) exposure was associated with higher cortisol levels (54%) in comparison to dim, red or blue light.	[73]
	Healthy volunteers	Increase in CAR (50%)<br>Increase in CR (AUC)<br>Persistent high cortisol	Blue (470 nm, 201 Lux) and Green (520 nm, 806 Lux) light promoted a greater cortisol awakening response in comparison to red light (635 nm, 235 lux).	[74]
	Healthy volunteers	Increase in CR (variable)	Meta analysis of 13 studies show a strong relation between severe caloric restriction (fasting) and high cortisol levels, but not for moderate or low caloric restriction.	[84]
Fasting	Obese patients	Increase in CR (Mesor; 11%)<br>Shift in acrophase (48 min)<br>Reduction in cortisol amplitude (52%)	1 day fasting increases the amplitude of cortisol production (11%) and shifts the acrophase (48 min) earlier.	[85]
	Healthy volunteers	Increase in CAR (37%)<br>Shift in acrophase<br>Persistent high cortisol<br>Presence of irregular peaks during sleep time	The effect of fasting and sleep pattern modifications on cortisol response was very variable across subjects. Additionally, the effects were not significant upon 4 weeks of constant fasting	[83,276]
Alcohol Consumption	Heavy and moderate alcohol consumers	Increase in CAR (37-89%)	Cortisol levels are positively associated with alcohol intake	[87]
	Alcohol consumers	Increase in CR	Cortisol levels increased by 0.18 μg/dL per drink per day	[89]
	Alcohol-dependent volunteers	Increase in CR	Cortisol levels were increased in intoxicated alcohol-dependent and no intoxicated subjects in acute alcohol withdrawal, in comparison to abstinent subjects	[90]
	Alcoholic Volunteers	Increase in CR	Cortisol levels were increased in alcoholic subjects during an experimental induced ethanol intoxication. The cortisol levels were positively correlated with the blood alcohol levels	[88]
	Healthy volunteers exposed to alcohol	Increase in CR upon alcohol consumption	Cortisol levels were higher in healthy subjects upon alcohol consumption in individuals with family history of alcoholism	[277]
High carbohydrate meals	Healthy volunteers	Increase in CR (AUC; 44%)	The cortisol response was increased after a high-carbohydrate meal but not under high protein or high fat	[97]
	Healthy volunteers	Increase in CAR (16%)	The cortisol awakening response was increased and the individuals consuming 2 or more sweetened beverages exhibit also increase fat depots	[99]
	Healthy volunteers	Increase in ACTH (5.6-fold)<br>Corticosterone (4-fold)	The cortisol amplitude and the duration of cortisol response was increase in a dose-dependent manner.	[96]

	Healthy volunteers	Increase in CR (AUC; 51%)	The cortisol peak and the duration of cortisol response were increased in a dose-dependent manner.	[95]
	Healthy volunteers	Increase in Acrophase (50%)	The cortisol peak and the duration of cortisol response was increased in male and females exposed to a physical or mental stressor when exposed to caffeine	[278]

CAR: Cortisol awakening response; CN: Cortisol at night; CR: Cortisol response (total cortisol during the day).
