# Supplementary material for: Impact of Modern Lifestyle on Circadian Health and Its Contribution to Adipogenesis and Cancer Risk
Source: Cancers (Basel). 2024 Nov 1;16(21):3706. doi: 10.3390/cancers16213706 (PMC11545514; doi:10.3390/cancers16213706)
Supplement: Supplementary file 1 [file cancers-16-03706-s001.zip › Supplementary Table S2.pdf]

Table S2: Expression and Dependency of Clock Genes in Different Cancer Types

Gene	Cancer type	Assay	Observation	Reference
ARNTL2	Breast cancer (BC; triple negative)	Transcriptomic analysis (GSEA)	High ARNTL2 expression correlates with higher immune cell infiltration, activation and function, specifically in the triple-negative phenotype	[279]
ARNTL2	Nasopharyngeal carcinoma (NPC)	Transcriptomic analysis (GSEA); RT-qPCR; functional assays in *ARNTL2* overexpressive / knockdown cell lines	*ARNTL2* is hypermethylated in NPC cell lines and consequently, mRNA expression and protein down-regulated. *ARNTL2* overexpression supressed NPC cells through cell cycle arrest; *ARNTL2* overexpression enhance the sensitivity to cisplatin	[280]
Bmal1	Osteosarcoma cell line	siRNA Knockdown	*Bmal1* knockdown ablates the cycling of most of the metabolites (129/137)	[281]
Bmal1	BC: low-grade, luminal (MCF7); high-grade, basal (MDA-MB-231)	RT-qPCR; Luciferase-reporter *Bmal1*-cell lines	Circadian oscillations (*Bmal1* activity) persist in low malignancy breast cancer cells but not in high malignancy	[282]
Bmal1	mAML	ChIP-Seq<br>ShRNA Knockout	Knockout decreases AML-BM load<br>Pharmacological inhibition of *Bmal1* expression diminishes AML cell viablity	[283]
Bmal1	mCRC	RT-qPCR	High *Bmal1* expression correlates with poor chemotherapy response; *Bmal1* SNPs corelates with poor overal survival in murine xenografts; *Bmal1* increases VEGFA and CRC proliferation	[284]
Clock	mAML	ChIP-Seq<br>ShRNA Knockout	Knockout decreases AML-BM load	[283]
Clock	SUP-T1 / RPMI8402 ALL cell lines	Sh Knockdown	Knockdown decreases cell viability, promotes apoptosis and inhibits the cell cycle; *Clock* knockdown improves the mice survival in T-ALL xenografts	[285]
CRY-1	hCLL	RT-qPCR; DNA methylation analysis	Hypermethylation of CpG islands in promoter region silences *CRY-1*. Methylation grade is proved to have a better pronostic and favourable outcome.	[285]
CRY-1				
CRY-1	Primary hCLL patients	RT-qPCR	High *CRY-1* expression correlates with high-risk leukemias (CD38+)	[286]
CRY-1	Primary hCLL patients	RT-qPCR	High *CRY-1* expression is related to a poorer survival	[287]
CRY-1	Osteosarcoma cell line	siRNA Knockdown	*CRY-1* knockdown promotes a bimodal distribution of the metabolite expression	[281]
CRY-2	Osteosarcoma cell line	siRNA Knockdown	*CRY-2* knockdown delays the metabolite cycling	[281]

Table S2: Expression and Dependency of Clock Genes in Different Cancer Types

PER2	Breast cancer: low-grade, luminal (MCF7); high-grade, basal (MDA-MB-231)	RT-qPCR; Luciferase-reporter Bmal-cell lines	Circadian oscillations (*PER2* activity) persist in low malignancy breast cancer cells but not in high malignancy	[282]
PER2	Myeloid leukemia	Transfection. siRNA Knockdown	Per2 expression induces cell cycle arrest and apoptosis induction	[288]
PER2	Oral squamous cell carcinoma	Sh Knockdown	downregulation of Per2 induce the cyclins expression and the cell proliferation	[289]
PER2	Acute myeloid leukemia	Per2 inducible stable cell line	Per2 expression results in growth inhibition, cell cycle arrest, and apoptosis	[290]
PER2	Chronic lymphoid leukemia	RT-qPCR	High Per2 expression is a predictor of a poor outcome (treatment free survival)	[287]
	Lymphoid and myeloid malignant cell lines	Transfection	Per2 inhibits cell proliferation	[290]
PER2	Chronic mieloid leukemia; cell line KCL22	RT-qPCR	CML patients decrease Per 2 expression; Per2 overexpression inhibits KCL22 cell proliferation	[289]
PER2	Lymphoid malignant cell lines	RT-qPCR	Per2 is down-regulated in large B lymphomas, and Pre-B cell acute lymphoblastic leukemias	[290]

hCLL: Human cronic lymphoblastic leukemia; mAML: murine acute myeloid leukemia; mCRC: metastatic colorectal cancer
